# Extensive thoracic vertebral and chest wall metastases as the initial presentation of breast cancer: a case report and literature review

**DOI:** 10.3389/fonc.2025.1632700

**Published:** 2025-07-17

**Authors:** Yergen N. Kenzhegulov, Daniyar K. Zhamoldin, Victor G. Aleinikov, Talgat T. Kerimbayev, Berik Zhetpisbaev, Serik Akshulakov

**Affiliations:** ^1^ Department of Minimally Invasive Neurosurgery, JSC “National Centre for Neurosurgery”, Astana, Kazakhstan; ^2^ Department of Spinal Surgery and Pathology of the Peripheral Nervous System, JSC “National Centre for Neurosurgery”, Astana, Kazakhstan; ^3^ Department of Pathology, JSC “National Centre for Neurosurgery”, Astana, Kazakhstan; ^4^ JSC “National Centre for Neurosurgery”, Astana, Kazakhstan

**Keywords:** breast cancer, bone metastases, thoracic spine, rib metastases, sternal metastases, spinal pain

## Abstract

Metastatic involvement of the bones remains the most common form of distant metastasis in breast cancer, largely due to the anatomical and functional characteristics of the thoracic spine, ribs, and sternum. These structures are notable for their high content of red bone marrow, rich vascularization, and their connection to Batson’s venous plexus, all of which facilitate their early involvement in oncologic dissemination. In certain cases, multiple metastases in the thoracic skeleton may represent the first and sole clinical manifestation of an undiagnosed malignant process, presenting considerable diagnostic challenges at the initial presentation in patients without a known oncologic history. A 60-year-old female patient presented with severe thoracic back pain. Imaging revealed multiple lytic lesions in the vertebral bodies of the thoracic spine, ribs, and sternum. The initial differential diagnosis included multiple myeloma and bone metastases. The patient underwent minimally invasive neurosurgical intervention involving spinal canal decompression and percutaneous vertebral biopsy. A percutaneous vertebral biopsy confirmed the presence of undifferentiated carcinoma. Subsequent PET-CT identified a metabolically active lesion in the breast, establishing the primary diagnosis, followed by the initiation of systemic therapy. This case, in conjunction with a review of the current literature, highlights the diagnostic complexity of presentations where pain is the sole initial symptom of an undetected malignancy. Such situations demand a high index of oncologic suspicion from the outset, timely application of advanced imaging modalities such as MRI and PET-CT, mandatory histological verification of affected regions, and strong interdisciplinary coordination to achieve accurate diagnosis and formulate a personalized treatment strategy.

## Introduction

Bone metastases represent the most common form of distant spread in breast cancer, occurring in 60-80% of patients with advanced-stage disease (1). The thoracic spine and ribs are particularly affected, which is attributed to their anatomical and physiological characteristics, including high vascularization, the presence of red bone marrow, and a direct connection to Batson’s venous plexus that facilitates the retrograde dissemination of tumor cells (2, 3).

Significantly, in 20-40% of cases, bone metastases are the first clinical manifestation of breast cancer, preceding the identification of the primary tumor, which significantly complicates diagnosis - particularly in patients presenting with isolated back pain and no prior oncologic history (4, 5). In such clinical scenarios, the use of extended imaging protocols, including magnetic resonance imaging and PET-CT, as well as targeted biopsy for morphological confirmation, is essential (6).

Current therapeutic strategies for bone metastases are based on systemic treatment, the use of antiresorptive agents, and local interventions - ranging from stereotactic radiosurgery to minimally invasive surgical procedures. In cases of spinal involvement, surgical decompression and stabilization can significantly improve neurological function and pain control, particularly when combined with stereotactic body radiotherapy (SBRT), as supported by recent clinical studies (7–10).

The presented case underscores the critical importance of this approach: extensive involvement of the thoracic spine and sternum served as the initial manifestation of previously undiagnosed breast cancer, highlighting the necessity of early oncologic suspicion, multidisciplinary collaboration, and timely neurosurgical intervention in the comprehensive management of such patients.

## Case description

### Patient information

A 60-year-old female patient was admitted to the Department of Minimally Invasive Neurosurgery at JSC “National Centre for Neurosurgery” with complaints of intense, persistent pain in the thoracic spine, radiating to the anterior chest wall, band-like in nature, accompanied by marked restriction of movement and episodic urinary incontinence exacerbated by pain. The symptoms had been progressively worsening over several months, beginning in December 2024 (**Table 1**).

**Table 1:** Timeline of the clinical case.

Date	Event
15.12.2024	Symptom onset: severe pain in the thoracic spine
03.03.2025	CT/MRI: preliminary diagnosis of multiple myeloma involving the thoracic spine and ribs
11.04.2025	Initial consultation and admission to JSC “National Center for Neurosurgery”
15.04.2025	Surgery performed: decompression at Th10-Th11 and percutaneous vertebral biopsy
21.04.2025	Histological report: metastasis of undifferentiated carcinoma
23.04.2025	Discharged from hospital
28.04.2025	PET-CT: primary lesion in the breast; bone metastases in Th1-Th12, sternum, and ribs
05.05.2025	Core needle biopsy of the breast: confirmed diagnosis of breast cancer; referred to oncology care

### Clinical findings

The patient reported intense, constant pain in the thoracic spine radiating to the anterior chest wall. The pain was band-like in character, aggravated by movement, and accompanied by significant restriction of motor activity. According to the patient, peak pain episodes were occasionally associated with transient urinary incontinence. Her posture was forced and antalgic. Palpation revealed marked tenderness at paravertebral points from Th1 to Th12, most pronounced in the paramedian zones. Percussion of the thoracic spinous processes elicited severe pain. A hyperesthetic segment was identified bilaterally from Th1 to Th12 in a dermatomal distribution, along with positive “girdle” signs. Neurological examination showed no focal deficits: the patient was alert, with intact cranial nerves, sensation, reflexes, and coordination. The Karnofsky Performance Status was 60-70, indicating limited self-care capacity and partial dependence on assistance. Laboratory tests revealed moderate normochromic anemia, leukocytosis, and thrombocytosis, with no abnormalities in biochemical or coagulation parameters.

**Figure 1:**
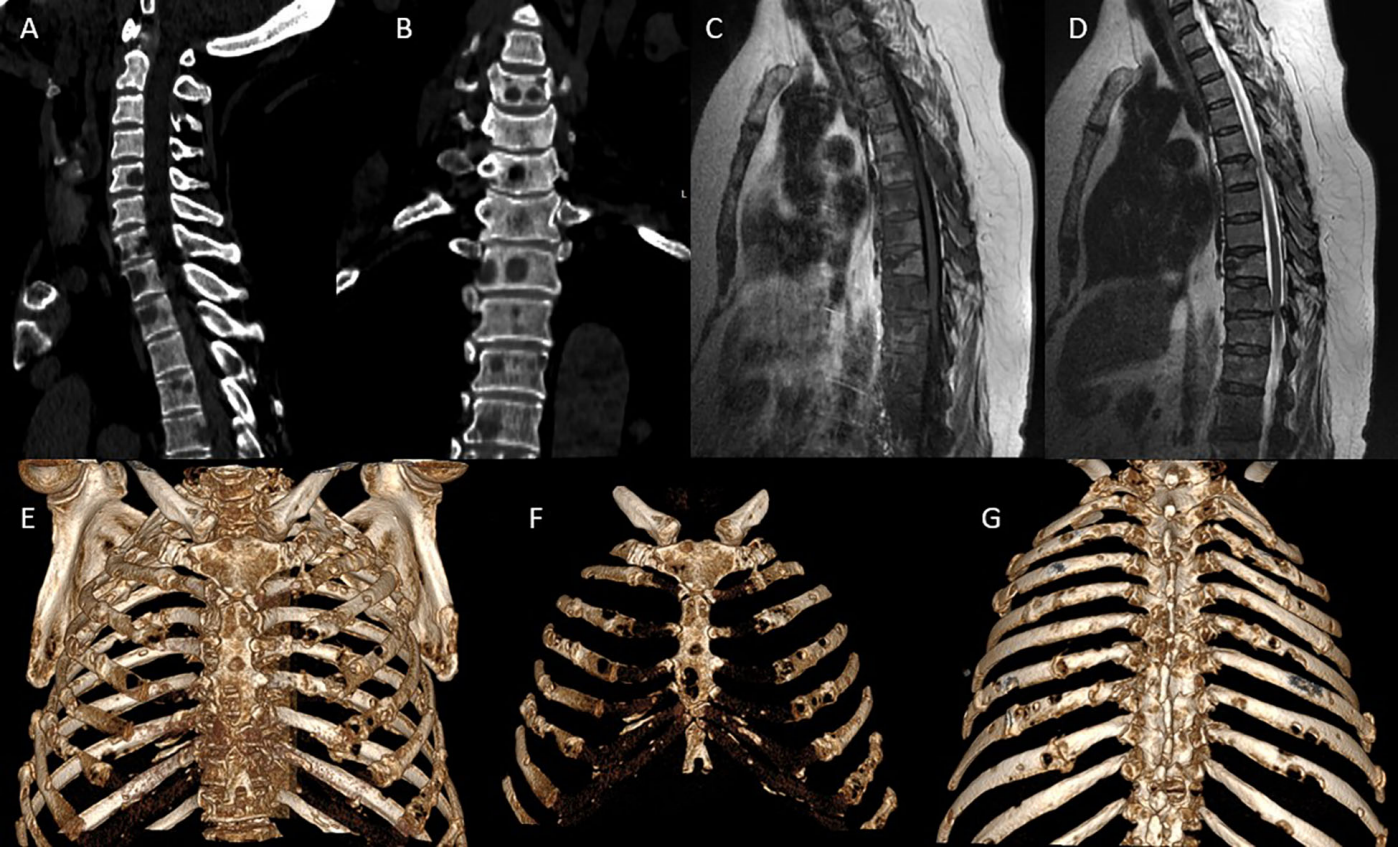
Preoperative CT/MRI imaging: **(A, B)** Multislice CT: sagittal **(A)** and frontal **(B)** reconstructions show multiple osteolytic lesions in the vertebral bodies from Th1 to Th12, with vertebral body collapse primarily at Th6-Th10, reduced intervertebral disc height, cortical bone destruction, and involvement of the posterior vertebral elements including the arches and facet joints. Extensive vertebral destruction is accompanied by lytic changes in the ribs and sternum. **(C, D)** Sagittal thoracic spine MRI: on T1-weighted images **(C)**, hypointense metastatic lesions are seen replacing the bone marrow of thoracic vertebrae. On T2-weighted images **(D)**, a distinct posterior paravertebral soft tissue component is evident, with spinal cord compression at Th10-Th11, associated with dural sac deformation and spinal canal stenosis. **(E–G)** 3D CT reconstructions of the thorax in various projections: anterior **(E)**, frontal **(F)**, and posterior **(G)** views demonstrate multiple metastatic rib lesions with lytic destruction, predominantly along the posterolateral arcs, as well as involvement of the sternum, including both the manubrium and body. Notable findings include thoracic cage deformation and signs of pathological fractures with resulting bone defects.

MRI of the thoracic spine revealed multiple metastatic lesions in the vertebral bodies from Th1 to Th12, against a background of degenerative-dystrophic changes. Multislice CT of the cervicothoracic spine demonstrated multiple areas of bone destruction and hypodense changes in the vertebral bodies and arches from Th1 to Th12, predominantly at the Th1–Th10 levels. The CT scan of the cervicothoracic spine included the breast fields; no abnormalities were detected in the breast tissue (no lesions seen in breast fields). An isolated destructive lesion measuring up to 1.1×0.7 cm was visualized in the cervical vertebral bodies. Intervertebral disc height was reduced, with signs of dehydration. Based on the combined MRI and CT findings - demonstrating multiple destructive foci in the vertebral bodies and arches from Th1 to Th12 with features of tumor infiltration - a preliminary diagnosis of multiple myeloma of the cervicothoracic spine was established (**Figure 1**).

### Diagnostic assessment

Based on the clinical presentation and characteristic osteolytic changes observed on initial imaging, the primary working diagnosis at the time of admission was multiple myeloma with predominant involvement of the cervicothoracic spine. The differential considerations included multiple metastases from solid tumors of unknown primary origin—most likely breast, lung, or thyroid cancer; less likely were primary bone tumors such as chordoma or osteosarcoma; and inflammatory etiologies, including spondylogenic osteomyelitis or tuberculous spondylitis, which were considered for exclusion. In the absence of a confirmed systemic diagnosis and without identification of a primary lesion, a decision was made to perform a percutaneous biopsy of the affected vertebra for histological and immunohistochemical verification. This procedure proved to be a pivotal step in clarifying the nature of the disease and guiding further therapeutic strategy.

### Intervention

During hospitalization, the patient received comprehensive treatment, including pharmacological pain management, thromboprophylaxis, supportive and symptomatic care, as well as neurosurgical intervention for both diagnostic and therapeutic purposes. On April 15, 2025, the patient underwent minimally invasive spinal microsurgery (MISS) using the Easy Go tubular endoscopic system and neuronavigation. The procedure included microsurgical decompression of the spinal canal at the Th10–Th11 level and a percutaneous biopsy of the Th10 vertebral body under fluoroscopic guidance. The surgery was completed without intraoperative complications, with an estimated blood loss of 50 mL. The surgical wound healed by primary intention, with intact sutures. Postoperative pain levels decreased to 2–3 points on the VAS (Visual Analog Scale).

### Follow-up and outcomes

**Figure 2:**
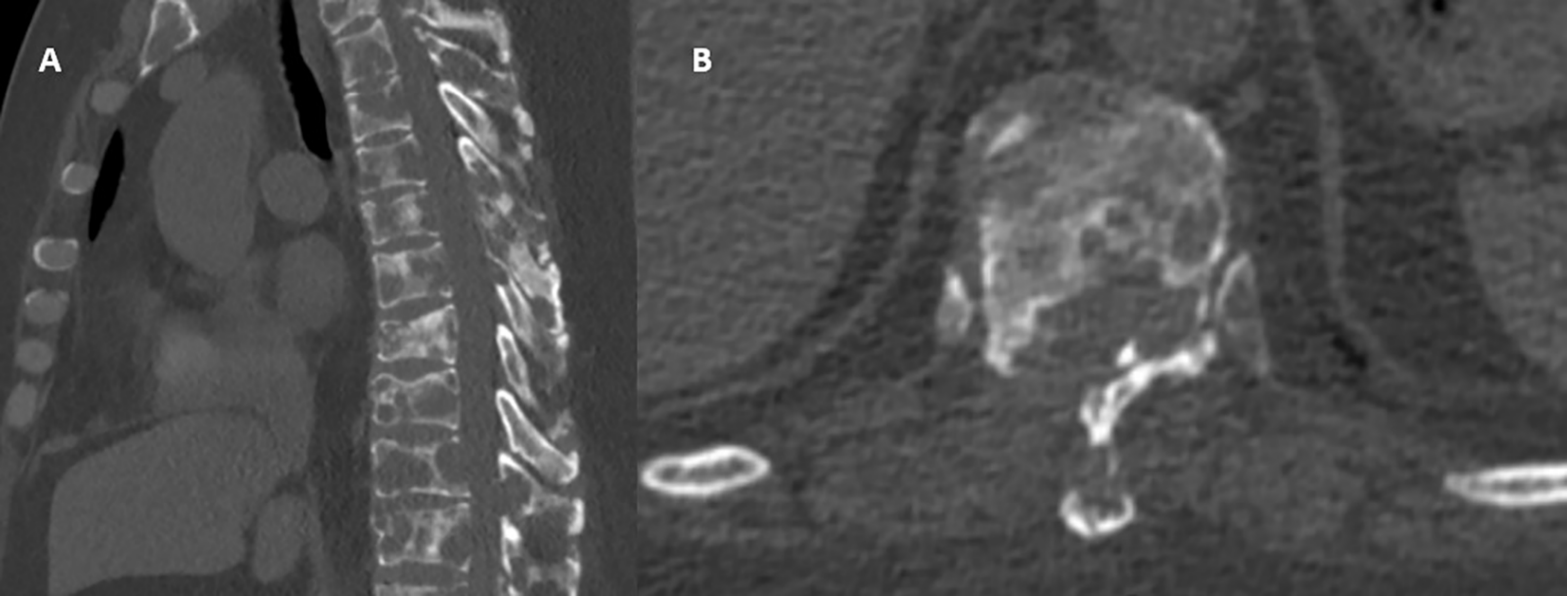
Postoperative CT imaging: **(A)** Sagittal view: confirmed decompression of the spinal canal at the Th10-Th11 level with partial removal of destructive masses and partial restoration of canal patency. Metastatic lesions persist within the vertebral bodies; however, compression is visually reduced due to surgical debridement. **(B)** Axial view: deformed Th10 vertebra with marked osteolytic destruction of the vertebral body and posterior wall; the spinal canal appears reconstructed. Paravertebral soft tissue components are present without evidence of volume progression.

**Figure 3:**
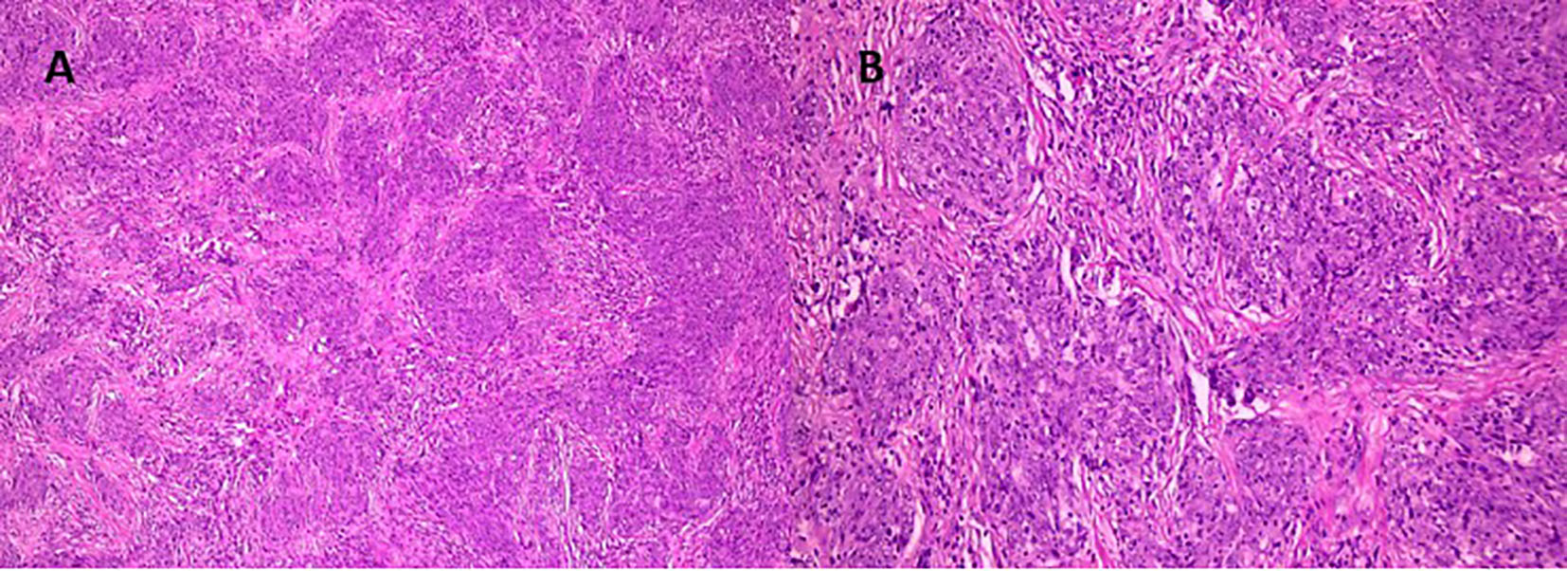
Histological examination: Hematoxylin and eosin-stained sections reveal tumor fragments displaying well-defined lobular and alveolar-like structures, along with areas resembling ductal formations [**(A)**, ×100; **(B)**, ×200]. The tumor cells are monomorphic, uniform, and cylindrical in shape, with pale perinuclear cytoplasmic halos and moderately basophilic nuclei. Focal zonal necrosis is observed in the centers of some duct-like structures, consistent with comedo-type necrosis. The stroma is fibrous, predominantly dense, with isolated sclerotic regions containing polymorphic hyperchromatic cells. At the periphery of the tumor nests, adherence to bony trabeculae is noted, indicating invasion into adjacent osseous tissue.

On postoperative day two, a CT scan of the chest was performed, confirming multiple osseous lesions, pathological rib fractures, and signs of a chronic bronchopulmonary process (**Figure 2**). Clinically, the patient demonstrated positive dynamics, including regression of pain (VAS 2-3), restored mobility, and stabilization of overall condition. By postoperative day eight, the patient was discharged in satisfactory condition with recommendations for further evaluation. Histological analysis of the vertebral body biopsy confirmed metastatic involvement by undifferentiated carcinoma (**Figure 3**). As part of the outpatient diagnostic workup to identify the primary tumor, a PET-CT scan was performed, which revealed a metabolically active area in the right breast and axillary lymph node. A core needle biopsy of the breast confirmed the primary tumor as right-sided breast cancer. The patient was referred for oncologic follow-up; staging is underway, and systemic therapy (chemotherapy/targeted therapy) is being selected based on the molecular profile.

## Discussion

Breast cancer (BC) remains the most common malignancy among women worldwide. As of 2020, more than 2.3 million new cases were diagnosed, with nearly 685,000 women dying from the disease (11). Particularly concerning is the situation in Asian countries such as Pakistan, where breast cancer is frequently diagnosed in women under the age of 50 and often at advanced stages, significantly reducing the likelihood of successful treatment (12). In the postmenopausal period, the risk of developing BC increases markedly, especially in the presence of factors such as obesity, absence of breastfeeding, and the use of hormone replacement therapy - findings supported by current clinical guidelines from the Japanese Breast Cancer Society (13). In some cases, the disease first manifests not with a palpable breast tumor but with already-established metastases. The presented case illustrates a rare and diagnostically challenging scenario in which the initial manifestation of BC was extensive metastatic involvement of the thoracic skeleton and spine. Such an atypical presentation can delay diagnosis and result in the initiation of treatment at the stage of disseminated disease.

Bone metastases play a pivotal role in the progression of breast cancer and have a profound impact on patient prognosis. The skeleton is the most common site of distant metastasis in metastatic breast cancer, with modern studies indicating involvement in up to 70% of cases. The presence of bone metastases significantly reduces survival outcomes: the median survival is approximately 36 months, while 5-year survival does not exceed 10% (14). Particular attention should be given to a rare yet severe scenario - symptomatic bone marrow involvement - which occurs in approximately 2% of cases, predominantly among patients with triple-negative breast cancer. This manifestation is characterized by pronounced cytopenia and necessitates a specialized approach to treatment and hematopoietic support (15).

In breast cancer, skeletal metastases most commonly affect the spine, ribs, sternum, and pelvis. Particular attention should be given to the thoracic spine (Th1-Th12), ribs, and sternum, as these anatomical regions are frequent and clinically significant targets of hematogenous dissemination. One of the key anatomical routes is Batson’s valveless vertebral venous plexus, which facilitates retrograde tumor spread to the thoracic region in response to changes in intrathoracic pressure (14). Up to 44% of all bone metastases are localized in the thoracic spine, making it the second most commonly affected region after the lumbar spine (16). The ribs rank among the top three most vulnerable skeletal structures, often involved in their arched segments rich in red bone marrow. Such involvement is typically accompanied by severe pain, restricted chest wall mobility, and a high risk of pathological fractures (17). Sternal metastases, most frequently affecting the manubrium, occur in approximately 40% of patients with chest wall metastases, particularly in those with primary breast or thyroid cancers. This can be associated with mediastinal compression, intense analgesic-resistant pain, and impaired respiratory function (18). In severe cases, respiratory failure may develop, necessitating thoracic stabilization through osteoplasty or cement fixation. Morphologically, lesions in the ribs and sternum may present as lytic, blastic, or mixed: lytic forms lead to bone destruction and predispose to fractures, whereas blastic lesions result in excessive but non-functional bone formation, complicating early diagnosis (19). According to Chen et al., 33% of patients with spinal metastases also have lesions in the thoracic vertebrae and ribs, while 66% exhibit extensive involvement of the posterior thoracic elements, requiring comprehensive treatment ranging from radiosurgery to cementoplasty and palliative procedures (16). In rare instances, widespread skeletal dissemination may extend to the bone marrow, leading to anemia and thrombocytopenia, which necessitates prompt initiation of chemotherapy with transfusion support. An especially rare but life-threatening presentation is total involvement of all thoracic vertebrae combined with metastases to the sternum and ribs - an imaging pattern indicative of advanced dissemination that requires urgent multidisciplinary intervention (15).

Clinical manifestations of metastases to the thoracic spine, ribs, and sternum in breast cancer are characterized by severe pain, restricted mobility, and respiratory impairment. Pain is the predominant symptom and is often constant, aching, or burning in nature, typically exacerbated by movement, deep inspiration, or coughing (20). In a meta-analysis by Han et al., the mean pain intensity at presentation reached 6.7 points on the VAS scale, rising to as high as 9 points in cases of sternal involvement (21, 22). Metastatic involvement of the thoracic vertebrae can result in spinal cord compression, presenting with progressive paraparesis, sensory deficits, and pelvic organ dysfunction, necessitating urgent neurosurgical intervention (23). Rib metastases are frequently associated with pathological fractures, even under minimal stress, leading to impaired respiration and reduced chest wall excursion (24). Sternal metastases, particularly in the highly vascularized manubrium, may cause acute localized pain, mediastinal compression, and respiratory failure. Some patients also present with pleuritis, dyspnea, and marked deterioration in general condition, requiring systemic analgesia, radiotherapy, and palliative measures. In severe cases, minimally invasive stabilization techniques - such as cement fixation, osteoplasty, and MRI-guided focused ultrasound - are employed to achieve effective pain control and improve quality of life (18, 22).

Modern diagnostics of metastases to the thoracic spine, ribs, and sternum in breast cancer relies on comprehensive imaging and laboratory evaluation of bone metabolism. The most informative modality is 18F-FDG PET-CT, which demonstrates high sensitivity (up to 93.8%) and specificity (99.1%) in assessing the thoracic wall and spine, especially when compared to conventional bone scintigraphy, which is less reliable in detecting lytic lesions (25). MRI remains the gold standard for early detection of bone marrow metastases, particularly in the thoracic vertebrae, outperforming CT in identifying lytic and mixed lesions. CT remains valuable for evaluating cortical bone integrity and assessing the risk of pathological fractures (26, 27). Despite advances in imaging and laboratory tools, biopsy remains essential when the clinical picture is ambiguous, histologic confirmation is required, or receptor status must be determined (25). When lesions are identified in the breast, primary histological verification via breast biopsy is preferable. In the absence of breast findings - i.e., when imaging shows no evidence of a primary breast tumor - a biopsy of the vertebral lesion is a justified first diagnostic step. However, access to the sternum and thoracic vertebrae is often technically challenging, and these procedures carry risks of complications and potential false-negative results. In clinically typical cases, when PET-CT and CT findings are characteristic and signs of systemic dissemination are present, biopsy may be deferred. Literature also discusses alternative diagnostic approaches such as PET/MRI - which combines anatomical precision with metabolic sensitivity - biochemical bone markers (e.g., BALP, P1NP, ICTP), and liquid biopsy, which allows for molecular assessment when bone tissue is inaccessible. Nonetheless, these methods currently do not replace traditional histological verification (26, 28).

When evaluating thoracic pain and bone lesions, it is crucial to consider a broad range of conditions that may mimic breast cancer metastases. The most common alternatives include primary bone tumors, spinal tuberculosis (Pott’s disease), and multiple myeloma (**Table 2**). To begin with, primary bone tumors such as osteoblastoma and giant cell tumor predominantly occur in younger individuals and are typically solitary lesions with slow growth and a less aggressive clinical course (37). Osteosarcoma, while capable of affecting the sternum and ribs, is more frequently diagnosed in adolescents and young adults. It often presents with a soft tissue component and tends to progress rapidly (38). Another important differential is Pott’s disease, the most frequent form of skeletal tuberculosis, which primarily involves the thoracic spine (Th7-Th12). It may present with chronic back pain, weight loss, low-grade fever, and signs of spinal cord compression. Imaging often reveals multiple vertebral body destructions with intervertebral disc involvement and the presence of paravertebral abscesses. Epidemiologically, tuberculosis should be considered in patients with HIV, recent migration history, or known exposure to tuberculosis (39). Furthermore, multiple myeloma - a malignant disease of the bone marrow - should be considered, particularly in patients over 60 years old. It is characterized by severe bone pain, anemia, fatigue, and predominantly osteolytic lesions in the spine and ribs (40). Unlike metastatic lesions, myeloma often presents with diffuse involvement without a significant soft tissue component and may yield false-negative PET-CT results. Notably, in elderly patients presenting with significant bone pain, anemia, and normal alkaline phosphatase levels, multiple myeloma should be excluded as a priority (41) (**Table 2**).

**Table 2:** Differential diagnosis of the case.

Pathology	Epidemiological Profile	Typical Localization	Lesion Characteristics	Clinical Presentation	Diagnostic Approaches
Breast cancer metastases	Women >40 years, known oncologic history	Thoracic spine, ribs, sternum	Multiple, osteolytic or mixed	Severe pain, spinal cord compression, pathological fractures	^18F-FDG PET-CT, MRI, confirmation of primary tumor
Osteoblastoma/GCTB	Young patients (10–35 years), no cancer history	Vertebral posterior elements, ribs (rare)	Solitary, well-demarcated	Localized pain, limited mobility, slow progression	CT/MRI, exclusion of multiple lesions
Osteosarcoma	Adolescents and young adults <30 years	Sternum, ribs, long bones	Solitary, destructive, with soft tissue extension	Progressive pain, swelling, rapid deformity, possible metastases	CT, MRI, aggressive radiographic features
Spinal tuberculosis (Pott’s disease)	Elderly, HIV+, immigrants, immunosuppressed	Th7–Th12 most common; cervical/lumbar less frequent	Involves 2–3 vertebrae in a row, disc involvement, paravertebral abscesses	Chronic back pain, low-grade fever, weight loss, neurological deficits	MRI, PCR (GeneXpert), microbiology, history of TB exposure
Multiple myeloma	Patients >60 years, more common in men	Spine, ribs, sternum, flat bones	Multiple, uniform, osteolytic	Spine/sternal pain, anemia, fatigue, hypercalcemia, normal ALP	Whole-body MRI (WB-MRI), CRAB criteria, serum electrophoresis, immunofixation

Data are based on sources (25, 26, 28–36). See the “References” section for full citations.

The management of bone metastases in breast cancer, particularly in the thoracic region, requires a strictly individualized and multidisciplinary approach. The cornerstone of treatment is systemic therapy: hormone therapy in HR-positive subtypes (especially in combination with CDK4/6 inhibitors), targeted therapy for HER2-positive tumors, and chemotherapy for triple-negative breast cancer. Combinations such as fulvestrant plus palbociclib have demonstrated an increase in median overall survival from 28 to 34.9 months (1, 42). In parallel, bone-modifying agents - bisphosphonates and denosumab - are essential for reducing the risk of skeletal-related events. According to Stopeck et al., denosumab decreases the risk of pathological fractures and spinal cord compression by 18% compared to zoledronic acid (43). Local control is achieved through radiotherapy. Stereotactic body radiotherapy (SBRT) provides local control in 94% of cases and complete pain relief in 36%, with vertebral stabilization achieved using doses of 24–30 Gy administered in 1–3 fractions (10, 44). Surgical intervention is indicated in the presence of spinal cord compression or neurological symptoms. Multicenter studies have shown that minimally invasive spinal surgery (MISS) results in clinical improvement in 83% of patients, with reduced blood loss and lower complication rates compared to open procedures (8, 9, 45, 46). In our case, given the extensive involvement of the thoracic spine and marked compression at the Th10 level, a decision was made to perform limited decompression at that level, followed by close stabilizing surveillance. Additionally, a percutaneous CT-guided vertebral biopsy was performed to achieve morphological verification and guide further systemic therapy planning under oncologic supervision. This approach allowed for minimized invasiveness while preserving both diagnostic and therapeutic efficacy.

Prognosis in patients with bone metastases from breast cancer largely depends on the tumor’s biological subtype, extent of disease, and the timeliness of initiating specialized treatment. In cases of isolated bone metastases, median survival may reach 30–36 months, whereas coexisting visceral involvement significantly reduces this figure (47). Functional status is another critical prognostic factor: pathological fractures, severe pain, and neurological deficits negatively impact quality of life and necessitate urgent intervention (48). The presented case underscores the importance of maintaining oncologic vigilance when encountering thoracic spinal pain, particularly in older women without evident signs of a primary tumor. Bone metastases may represent the first clinical manifestation of breast cancer, shaping both the diagnostic pathway and therapeutic approach. In this case, targeted decompression at the site of neurological compromise and CT-guided percutaneous biopsy not only alleviated symptoms but also enabled morphological verification essential for guiding systemic treatment. Comprehensive management - including antiresorptive agents, stereotactic radiosurgery, and multidisciplinary care - remains the cornerstone of effective palliative control. When treatment strategy is properly structured, even in the context of extensive skeletal dissemination, it is possible to achieve clinical stabilization, improved quality of life, and preservation of patient independence.

This case highlights a broader diagnostic dilemma that may arise in similar scenarios. While it underscores the importance of maintaining oncologic vigilance and utilizing a stepwise diagnostic algorithm, it also raises the following clinical question: in patients who present with isolated lytic skeletal lesions but without radiologic evidence of a primary tumor in the breast, to what extent is it appropriate to pursue breast biopsy? This diagnostic consideration has not been extensively addressed in the literature and merits further study. In such contexts, biopsy of the vertebral or skeletal lesion often becomes the initial step in diagnostic confirmation. However, the optimal sequencing of biopsy procedures - particularly in patients with no palpable or visible breast abnormalities - remains uncertain. Further research is needed to assess whether early breast biopsy, even in the absence of imaging abnormalities, could potentially accelerate diagnosis or reduce the need for more invasive procedures such as spinal biopsy.

## Conclusion

This clinical case demonstrates that isolated osteolytic lesions in the thoracic skeleton may serve as the initial and sole manifestation of breast carcinoma. In the absence of a known oncologic history, it is the physician’s oncologic vigilance, a multidisciplinary strategy, and a stepwise diagnostic algorithm - from advanced imaging through targeted biopsy - that collectively enable timely and accurate diagnosis. Early confirmation of metastatic disease is paramount for the prompt initiation of appropriate therapy and for optimizing patient prognosis. Furthermore, this case highlights an under-addressed diagnostic dilemma in the literature: whether a breast tissue biopsy should be pursued in patients who present with suspicious osseous lesions yet lack radiological or clinical evidence of a primary breast tumor. This question merits further investigation and the establishment of evidence-based guidelines.
